# Participatory Prototyping of a Tailored Undetectable Equals Untransmittable Message to Increase HIV Testing Among Men in Western Cape, South Africa

**DOI:** 10.1089/apc.2021.0101

**Published:** 2021-11-05

**Authors:** Philip J. Smith, Dvora L. Joseph Davey, Laura Schmucker, Cal Bruns, Linda-Gail Bekker, Andrew Medina-Marino, Harsha Thirumurthy, Alison M. Buttenheim

**Affiliations:** 1 The Desmond Tutu HIV Centre, Institute for Infectious Disease and Molecular Medicine, Faculty of Health Science, University of Cape Town, Cape Town, South Africa.; 2 Department of Epidemiology, Fielding School of Public Health, University of California Los Angeles, Los Angeles, California, USA.; 3 Division of Epidemiology and Biostatistics, School of Public Health and Family Medicine, University of Cape Town, Cape Town, South Africa.; 4 Medical Ethics and Health Policy, Perelman School of Medicine of the University of Pennsylvania, Philadelphia, Pennsylvania, USA.; 5 Center for Health Incentives and Behavioral Economics, Medical Ethics and Health Policy, Perelman School of Medicine of the University of Pennsylvania, Philadelphia, Pennsylvania, USA.; 6 Matchboxology, Kalk Bay, Cape Town, South Africa.; 7 Division of Men's Health, Desmond Tutu HIV Centre, University of Cape Town, South Africa.; 8 Department of Psychiatry, Perelman School of Medicine of the University of Pennsylvania, Philadelphia, Pennsylvania, USA.; 9 Department of Family and Community Health, School of Nursing, University of Pennsylvania, Philadelphia, Pennsylvania, USA.

**Keywords:** medication adherence, prevention, antiretroviral, HIV, undetectable, U=U

## Abstract

Daily antiretroviral therapy (ART) suppresses viral replication, rendering HIV undetectable through viral load (VL) testing. People living with HIV (PLWH) who have an undetectable VL cannot transmit HIV to sexual partners or through giving birth, a message commonly referred to as U = U (undetectable equals untransmittable). To increase knowledge and understanding of U = U among men, who have poorer HIV testing and treatment outcomes than women, we engaged men from high HIV burden communities in Cape Town in two interactive human-centered design cocreation workshops to develop local U = U messaging for men. Two trained workshop facilitators, explained the U = U message to 39 adult men (in two separate workshops), and asked them how to effectively communicate U = U to other men in the local language (isiXhosa). Participant-designed messages sought to inform men about U = U to help assuage fears of testing HIV positive (by removing the stigma of living with HIV and being a vector of disease), and to explain that ART enables PLWH to live normal healthy lives, making HIV “untransmittable” to sex partners. Participants' messages emphasized that when virally suppressed, “*I cannot spread HIV to the other person*” and “*(the pill) keeps on killing the virus so I can live a normal life for the rest of my life.*” Men cocreated simple local U = U messages to address fears of testing HIV positive and emphasizing ART's positive effects. Cocreated tailored messaging may reduce stigma associated with living with HIV and improve the uptake of HIV testing and treatment among South African men. This study was registered at clinicaltrials.gov under NCT04364165.

## Introduction

South African males, compared with their female counterparts, are less likely to test for HIV (58% vs. 64%), know that they are living with HIV (78% vs. 89%), initiate antiretroviral therapy (ART) (53% vs. 64%), and achieve viral suppression (43% vs. 58%).^1–5^ In South Africa, ART scale-up has reduced HIV-related mortality and, since 2012, life expectancy has improved from 63.6 to 66.7 years in 2017.^5^ The difference between HIV testing in males and females was most pronounced in the 15–24-year-old age group where 76% of males were unaware of their HIV-positive status compared with 35% of females.^5^ HIV prevalence peaked for men (25%) in the 45–49-year-old age group, compared with 35–39 years (39%) for women. These disparities result in higher morbidity and mortality among men living with HIV.^6,7^ High disease burden communities require interventions that will increase testing among men as well as ART uptake and retention among men living with HIV, thus increasing viral load (VL) suppression at the individual and community levels.^8,9^

Daily ART suppresses viral replication, rendering HIV undetectable through VL testing within 24 weeks.^10^ HIV-positive individuals with an undetectable VL cannot transmit HIV to sexual partners or through giving birth, a message commonly referred to as U = U (undetectable equals untransmittable, or treatment as prevention). U = U messaging has been implemented worldwide, with awareness highest among men who have sex with men, followed by women who have sex with men.^11^ In addition to the benefits of VL suppression, U = U has been associated with increased willingness to take up treatment, reduced feelings of stigma, and increased willingness to disclose HIV status.^12^ In a recent study, rural South African men were aware of the positive effects of ART on “regaining one's health,” improving appearance and increasing longevity; however, few of the participants were aware of the preventive effects of HIV treatment.^13^ Moreover, once the participants were made aware of the effects on transmission, they offered that knowing this would motivate men to test for HIV and could improve ART adherence.

U = U messaging has been recommended for endemic settings such as South Africa.^14^ However, there is limited development of messages to communicate the U = U message to South Africans. Given that South African men have poorer HIV-related outcomes, we cocreated U = U messages with South African men with the expressed purpose of increasing the uptake of HIV testing and treatment (re-)initiation among their peers. The effectiveness of these messages was tested in a cluster-randomized trial reported elsewhere,^15^ where men who received the U = U invitation had almost double the odds of HIV testing compared with men who received a standard invitation promoting free testing. This article describes the process used to develop the U = U messages for men.

## Methods

### Setting and participants

The study was conducted in the Klipfontein Mitchells Plain (KMP) District in Cape Town, South Africa. KMP has a high HIV disease burden and a high population density. A trained recruiter from Gugulethu used convenience snowball sampling to recruit men and their peers, aged 18–49 years, from high-traffic locations in the district. Gugulethu is based in the KMP District, which is a periurban limited resource area 10–15 min drive outside of Cape Town. The University of Cape Town IRB reviewed and approved the study (reference: HREC ref 750/2019). Participants gave their written informed consent, including dissemination of the content they generated, and pictures and videos taken of their participation. The study was registered at clinicaltrials.gov under NCT04364165.

### Human-centered design workshops

The workshops were designed to develop a U = U message using a participatory human-centered design (HCD) framework.^16,17^ There is increasing recognition that intervention design should incorporate viewpoints from the target audience to enhance collaboration, improve acceptability, and increase uptake.^18,19^ This participatory research included two workshops from a population of men that may potentially be recipients of the intervention. The trained recruiter invited men from KMP to participate in a 1-day interactive HCD cocreation workshop about HIV treatment, HIV testing, and barriers to both. HCD has been used to cocreate tailored products and services with end users for whom such products and services are intended.^17^ The participatory process used in the HCD method has been used for HIV testing, prevention, and treatment services.^20^ Two trained facilitators presented the theory behind the U = U message to the participants with the goal of developing a U = U message for their peers.

### Process

**FIG. 1.**
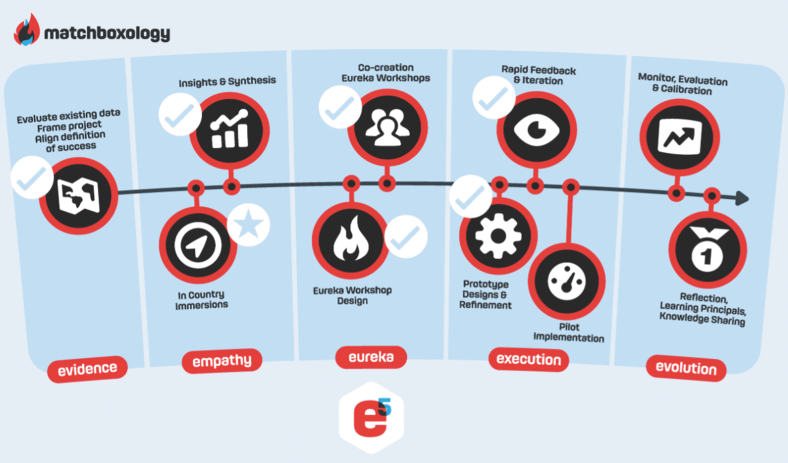
Human-centered design workshop process.

**FIG. 2.**
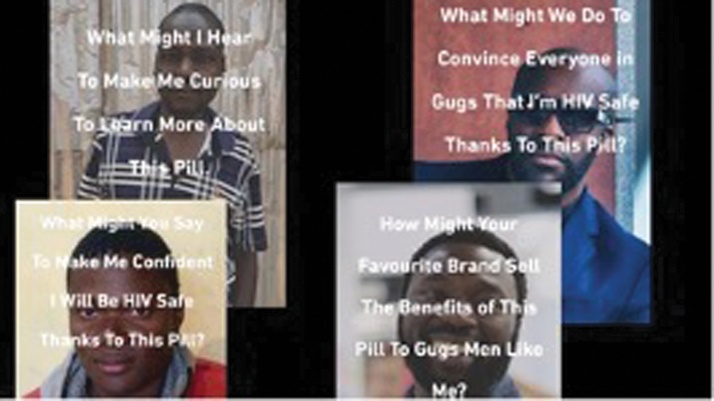
Posters presented to the men to initiate discussion about U = U and how they may translate it to their peers in their community.

The workshops were designed to develop a locally relevant and resonant U = U message. After workshop participants provided informed consent, two trained facilitators presented, in layman's terms, the scientific rationale and evidence substantiating the U = U message (Fig. 1). The facilitators presented the U = U message to participants, stating that people living with HIV (PLWH) who take ART daily will reduce the virus to the extent that they will not infect their sexual partners. The goal was to detraumatize HIV infection with a clear picture of a normal future that could mitigate the fears of testing for HIV and diagnosis with HIV.^21–23^ The facilitators then explained that the workshop's purpose was to elicit insights from the men and cocreate U = U messaging for the local context. Specifically, the facilitators asked (1) what might I hear to make me more curious about antiretrovirals (ARVs), (2), what might we do to convince everyone in my community that I am HIV safe (e.g., cannot transmit the virus) thanks to this pill, (3) what might we say to make *you* confident that I am HIV safe thanks to this pill, and (4) how might your favorite brand sell this pill to men like you? In addition to stating these questions out loud, they were also displayed on posters in the workshop venue (Fig. 2). Workshop participants were then facilitated through a series of HCD group exercises designed to cocreate a face-to-face presentation of this information (i.e., a 1-min “sales pitch”) that they believed would resonate with their community peers in language, tone, and structure.

### Analysis method

**FIG. 3.**
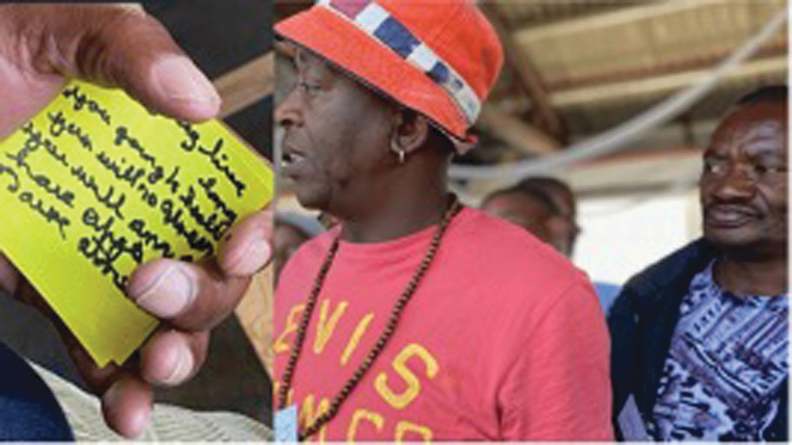
Images from participants in the workshops.

Men presented their initial suggestions for the U = U message as sales pitches and received group feedback. These pitches were recorded and transcribed by P.J.S. We coded the transcribed responses presented in the workshop (P.J.S and D.L.J.D.). We used a thematic analysis framework to code and analyze the results.^24^ Based on the group feedback, men refined and represented their sales pitch, which was audio recorded and transcribed (Fig. 3).

Final pitches were ranked by participants in order of perceived impact and potential to change behaviors and attitudes of men. Through a facilitated feedback session, men highlighted specific benefits about U = U that they felt resonated with them and with men in their community. Lastly, participants were reimbursed (R150 ZAR, ∼11USD, ∼90% of daily minimum wage) for their time, transportation, and participation in the 3–4-h workshop.

### Prototyping

Transcribed sales pitches were compiled to develop a script that was ∼60 sec. In a second workshop, the outcomes from the first workshop were presented to men to evaluate their understanding and suggest improvements to the prototype message. The discussions and the pitches were mostly in isiXhosa and were transcribed and translated into English. After the second HCD workshop, the messaging was fine-tuned with technical experts, including local behavioral scientists and researchers who have worked in HIV treatment and prevention delivery in South Africa (P.J.S., D.L.J.D., and L.G.B.), from the Desmond Tutu Health Foundation and University of Cape Town. The script was tested among 11 men (*n* = 7 from the KMP District and *n* = 4 HIV counselors from a mobile HIV testing clinic), after which a brief 10-question survey was administered to assess message comprehension, where opinions were sought, and suggestions invited for improving the message. The final message was summarized on printed invitation cards and delivered verbally by male peers to intervention participants in a cluster-randomized trial.^15^

## Results

We enrolled 39 adult men from the local community. Men shared their understanding of U = U and their message for other men in their community. The first theme was to explain the various benefits of taking ART. Men wanted others to know that ART improves the health of PLWH and their immune system functioning, leading to a healthy long life. Men related the theme of longevity to VL suppression, stating:

*The more you take the pill consistently, the longer you will live.*

*This medicine boosts your body and the immune system.*

The next theme that men spoke about was disclosure and stigma related to living with HIV. They felt that the U = U message would transform their identity and empower them to disclose their status. Since an HIV diagnosis was associated with worry about disclosure and deteriorating health, men stated that the U = U message would address those concerns. The participants said that the message could change the way that men saw themselves by reducing worry about a range of issues connected with concealing one's HIV-positive status:

**Table 1.** Prototyped U = U Testing Invitation Script

U = U intervention invitation message
1. Hi! My name is (peer promotor's name), and I work for the Desmond Tutu Centre. 2. Do you know “iMpilo”? [*Note: impilo means health in Xhosa and is a play on words as we are referring to ART pill in this message*] a. iMpilo is the latest mahala ARV pill that you take once a day if you are infected with HIV. 3. Did you know that iMpilo protects you *from getting sick* because it reduces HIV in the body—so much so that you can't infect your partner and family? a. This is called U = U. 4. It protects you even if you don't use a condom. 5. Even if you're drinking. Did you know that? 6. So in no time you're “Ugrand” [meaning, strong/courageous] by protecting your partner(s) and family. a. Your life stays the same and doesn't change. 7. You and I can show our kasi [community] how to do this thing one by protecting our kasi “Khusela ikasilam” [protecting our community] 8. Tutu Tester (point to location) can quickly tell you your HIV status and iMpilo for mahala [for free]. a. Take this invitation with you. See you there!

ART, antiretroviral therapy; ARV, antiretroviral.

*You are able to disclose your status now because at least you won't be infectious.*

*No more fear, no more humiliation, no more stigma, no more loneliness, no more failure.*

Key to the U = U message was the opportunity to renew the hopes of men living with HIV (MLHIV) for a family life, having children, rearing a family, and social interactions, as these opportunities and aspirations seemed less possible in the aftermath of a diagnosis with HIV. Participants stated that the U = U message could communicate that these aspirations were still attainable, stating:

*You can grow your family without the fear of HIV.*

Participants wanted to reintroduce the pill to ensure that men understand the safety of ART, and the reduction of transmission to partners. The theme of safety had the following subthemes; safe sex, partner/family protection, and ART pill safety. A number of participants highlighted that ART reduces VL, which would reduce HIV transmission to sex partners:

*It reduces the viral load in your body, that if only you have been tested positive, I am not saying that you are. When you are taking this tablet, you cannot spread the virus to the other person.*

*If you take this pill everyday, it 100% guarantees that you will be safe.*

*This pill doesn't make you feel weak after taking it.*

Men offered that it was a high priority to communicate that the key U = U message is that while on ART, men are protecting their partners from HIV infection. This was extended to family members, with men stating that ART would protect unborn children from HIV infection.

*Have a safe sex life and grow your family without the threat of HIV.*

*The pill can boost your self-esteem and it can prevent an unborn child from contracting HIV.*

Participants highlighted that along with U = U messaging, men should know that ART is easy to use and free. A participant who was not familiar with ART as a once-a-day pill said that when he became aware of the simplified regimen, “… *it was music to my ears.*” Similarly, others commented on the convenience of taking one pill a day and any time during the day.

*It's convenient to use it because it's just one pill a day.*

Participants noted that it was important to highlight that ART would have a financial cost for men, *“Don't buy life, you've got the pill for free.”*

Participants also highlighted being able to drink alcohol while on ART (which many did not know about before the workshop). Men expressed relief at the reduced social impact of ART, including the option to drink alcohol. Coupled with the assurance that an undetectable VL eliminated transmission, these two messages reduced anxiety around social behaviors that were central to men's identity and the enjoyment of social relations.

*You can drink it with alcohol.*

*You can use it even when you are drinking.*

In terms of recommendations for how to communicate U = U messages, men recommended that the message should be offered in multiple mediums, locations, and activities. Participants recommended using various activities to raise awareness among men. These activities included outreaches in taverns and shebeens (bars), social media marketing, community support groups, workshops, radio advertisements, songs, school visits, and men's summits. Men offered places and locations where awareness raising could take place, including mobile clinics, shopping malls, public transport, prisons, churches, billboards, music events, clinics, and clubs.

### Pitches

The men used the insights that they had generated and shared with the workshop to create and deliver a U = U messaging sales pitch to their peers. Following are examples of translated pitches:

*It's a new tablet that has just come to the market, it is very helpful. Okay, I am also using this pill, it's new on the market. Otherwise, I am also HIV-positive brother, when I heard about this pill, I decided to use it. I have used it the way it was prescribed to me. It has raised me from the dead. So, can I explain it to you? Thank you for giving me your time brother. This pill is very helpful because I was bed bound and clueless, and then another guy came to explain to me about this tablet. I tried it while I was bed bound, so it helped me to get up. If you are interested on this tablet, I would like you to take it and try it on yourself and see how it works.*

*Most of the time this tablet is perfect for your health, but I would recommend that when you have time get tested so that you know your HIV status. Thank you very much for your time.*

*Rapping: “Umtholampilo (the pill) keeps on killing the virus in my body, so you can also take this Umtholampilo (the pill) so that you do not get infected.”*

*I explained to the potential customer (person) that I have HIV and this pill helped me, by the way if you do not want to use a condom you cannot use it when you use this pill.*

*The medication always kills so much virus in me. So, I will not infect you. No stress no worries, no pain and stress and no miseries.*

*That is me, I won't be able to infect you with the disease.*

*The pill keeps killing so much virus in me so I can't infect you baby.*

### Intervention prototype

Insights and pitches were iteratively developed into a simple script in isiXhosa that could be used in a brief face-to-face encounter to invite men to test. After the development of the message, it was tested in a cluster-randomized trial at five community HIV testing sites.^15^

## Discussion

We used participatory prototyping and HCD to translate the U = U concept into a brief upbeat local message that was delivered by male peers to invite men to community HIV testing services. Men have reported being fearful of HIV, and ART is often associated with complex regimens, side effects, and the associated hassle of visiting a clinic.^25^ In the workshop, men wanted to shift the emphasis of ART to highlight the beneficial outcomes of taking treatment. To increase HIV testing uptake and ART use, workshop participants designed messages focusing on the preventive benefits of taking ART and the ways in which U = U messaging can reduce fears of an HIV diagnosis and disclosure. Integral to the message was that men were aware that taking ART was easier than it had been in the past, and that treatment did not have to impact on social behavior, such as drinking alcohol with friends, having a partner, and raising children. The messages also highlighted that that daily ART reduced HIV VL, which translated into being healthy, living a long life, while, at the same time, preventing the spread of HIV to sex partners.

Stigma and denial remain significant problems in HIV endemic communities in South Africa.^26^ Men are oftentimes disinclined to screen for illnesses, such as HIV, that are associated with wasting or death. A common psychological barrier to testing was echoed in our workshops: men do not want to find out whether they have a dreaded disease like HIV because they think they will worry themselves to death. This is a particularly challenging barrier given that many men become HIV positive when they are young and feel healthy or are asymptomatic. In addition to self-stigmatizing attitudes,^27,28^ community stigma^29^ can cause men to delay taking up HIV-related services. This presents an opportunity to explore strategies that will reduce stigma in the service of lowering barriers to access and improving the experience of HIV care. Tailored messaging about the benefits of ART on reducing HIV transmission (U = U) may circumvent the stigma and denial associated with testing and initiating ART.

Mental models about the meaning of an HIV diagnosis and the ability of ART to promote a long and healthy life have not been updated for the realities of modern ART. Although the U = U messages we developed emphasize these new mental models, this has not been the case for public health campaigns in South Africa to date. The majority of men in our workshops, who were not HIV positive and thus experienced with the latest ART, believed that treatment still required taking many pills per day (as it had a decade ago), had severe and numerous side effects, and offered only moderate treatment success.

Limitations of our study include generalizability of the messages. We sampled participants from high HIV burden communities in Cape Town. It is not known whether insights and pitches generated from this codesign process will generalize to men in other communities in South Africa or in other countries. Although we generated prototype designs for U = U-informed health communications interventions, further field testing to assess their effectiveness is required.

Ending the HIV epidemic is dependent upon a cascade that identifies and tests all at-risk populations, links PLWH to care, promotes treatment initiation and adherence, and results in sustained VL suppression—especially focusing on men in South Africa. U = U messaging that leverages what the community knows and thinks about HIV can motivate men to seek HIV testing, and be used in multipronged health promotion communications packages to improve men's entry into the HIV prevention and treatment cascades. Tailored HCD messaging to promote HIV testing and early ART initiation may be a valuable addition to encourage health-seeking behavior, and facilitate retaining clients over time. Accordingly, future studies should investigate the impact of a tailored U = U message on improving HIV cascade outcomes for men, from testing uptake to VL suppression.
